# Phylogenetic Analysis of Chikungunya Virus Eastern/Central/South African-Indian Ocean Epidemic Strains, 2004–2019

**DOI:** 10.3390/v17030430

**Published:** 2025-03-18

**Authors:** Alessandra Lo Presti, Claudio Argentini, Giulia Marsili, Claudia Fortuna, Antonello Amendola, Cristiano Fiorentini, Giulietta Venturi

**Affiliations:** Department of Infectious Diseases, Istituto Superiore di Sanità, 00161 Rome, Italy; claudio.argentini@iss.it (C.A.); giulia.marsili@iss.it (G.M.); claudia.fortuna@iss.it (C.F.); antonello.amendola@iss.it (A.A.); cristiano.fiorentini@iss.it (C.F.); giulietta.venturi@iss.it (G.V.)

**Keywords:** CHIKV, genetic diversity, phylogeny, evolution, early warning, genomic surveillance

## Abstract

CHIKV infection is transmitted by *Aedes* mosquitoes spp., with *Ae. aegypti* considered as the primary vector and *Ae. Albopictus* playing an important role in sustaining outbreaks in Europe. The ECSA-Indian Ocean Lineage (IOL) strain emerged in Reunion, subsequently spreading to areas such as India, the Indian Ocean, and Southeast Asia, also causing outbreaks in naive countries, including more temperate regions, which originated from infected travelers. In Italy, two authocthounous outbreaks occurred in 2007 (Emilia Romagna region) and 2017 (Lazio and Calabria regions), caused by two different ECSA-IOL strains. The phylogenetics, evolution, and phylogeography of ECSA-IOL-CHIKV strains causing the 2007 and 2017 outbreaks in Italy were investigated. The mean evolutionary rate and time-scaled phylogeny were performed through BEAST. Specific adaptive vector mutations or key signature substitutions were also investigated. The estimated mean value of the CHIKV E1 evolutionary rate was 1.313 × 10^−3^ substitution/site/year (95% HPD: 8.709 × 10^−4^–1.827 × 10^−3^). The 2017 CHIKV Italian sequences of the outbreak in Lazio and of the secondary outbreak in Calabria were located inside a sub-clade dating back to 2015 (95% HPD: 2014–2015), showing an origin in India. Continued genomic surveillance combined with phylogeographic analysis could be useful in public health, as a starting point for future risk assessment models and early warning.

## 1. Introduction

Chikungunya virus (CHIKV) is a single-stranded positive-sense RNA virus belonging to the genus *Alphavirus* of the *Togaviridae* family. CHIKV infection can cause high fever, rash, myalgia, and arthralgia, which can lead to chronic pain and disability [1]. It is transmitted by *Aedes* (*Ae.*) mosquitoes spp., with *Ae. aegypti* considered as the primary vector, and *Ae. albopictus,* which has acquired increasing importance in recent decades for its role in sustaining outbreaks in Europe [2,3,4,5,6,7].

Driven by human movement and climate trends, the distribution of these mosquitoes is expanding, along with the arboviruses they transmit [8]. Particularly, *Ae. aegypti* probably originated in Sub-Saharan Africa and established itself in most of the world’s tropical and subtropical regions owing to globalization and human activities [9,10]. Meanwhile, *Ae. albopictus*, native to Southeast Asia, the islands of the Indian Ocean, and the Western Pacific [11,12], recently expanded in Africa, the Americas, and more temperate regions such as Europe, where it is spreading more and more. CHIKV causes explosive outbreaks before apparently disappearing for periods of several years to decades [13,14,15,16].

After the first CHIKV isolation in Tanzania in 1952, three distinct globally dispersed CHIKV genotypes were identified and named “Western African”, “Asian”, and “Eastern/Central/South African” (ECSA) [17,18].

Since 2004, ECSA and Asian genotypes have been responsible for large outbreaks in the Indian Ocean, Asia, the Pacific, and the Americas.

An ECSA strain emerged during the outbreak in Kenya in late 2004, with subsequent expansion to Reunion Island, where the ECSA-Indian Ocean Lineage (IOL) strain emerged and subsequently spread to areas such as India, the Indian Ocean, and parts of Southeast Asia, leading to over 6 million estimated cases of CHIKV disease over time [19,20,21]. International air travel facilitated the CHIKV expansion with travelers, importing cases into naive countries, including more temperate regions in Europe and the United States, and most importantly, seeding autochthonous transmission of the virus in temperate areas, including Italy in 2007 [2] and France in 2010 [22]. More recently, a second autochthonous outbreak in Italy occurred in 2017, in two different regions (Lazio and Calabria), caused by a ECSA-IOL strain different from that of 2007 [3,4].

The CHIKV genome encodes four non-structural proteins (NSP 1–4) and three structural proteins (C, E1, and E2). In particular, the viral-surface envelope-anchored protein E1 performs membrane fusion functions, playing an important role in efficient replication in the insect vector. Mutations in the E1 gene are important for the adaptation of CHIKV to different vector species. The A226V mutation (E1 protein), which has been detected in the virus strain responsible for the 2007 Italian outbreak but not in the 2017 outbreak, has been demonstrated to improve the adaptation of the virus to *Ae. albopictus* [6,23].

The CHIKV ECSA strains expanded and generated outbreaks in Europe and are well adapted to *Ae. albopictus*; for these reasons, it is important to study the molecular evolution and phylogeny of CHIKV.

Genotypic and phylogeographic analysis are important to understand the origin and spread of CHIKV during epidemics and the diversity of circulating strains for the risk assessment and implementation of effective control measures. The genomic surveillance of pathogens with epidemic potential can be a useful tool for risk assessment and early warning. Phylogeography can contribute to these purposes by allowing one to make use of the sequence data available in databases, accompanied by appropriate metadata (i.e., the place of acquisition of the infection, and the collection date), in order to determine the space-time evolution of a pathogen.

In this study, we aimed to investigate the phylogeographic spread and mean evolutionary rate of ECSA-IOL-CHIKV strains causing outbreaks in Italy in 2007 and 2017, to better understand where and when these outbreaks originated and CHIKV circulation. Phylogeographic analysis can be also useful as a predictive model for risk assessment and for future events, and it may be applicable to other pathogens.

Finally, we estimated the CHIKV-E1 phylogenetic relationships and clustering, and the place of occurrence of the mutations related to adaptation to *Ae. albopictus*, which caused outbreaks in temperate areas, particularly in Italy.

## 2. Materials and Methods

### 2.1. CHIKV Sequence Dataset

The dataset consisted of 258 CHIKV E1 sequences; all of them belonged to the ECSA-Indian Ocean (ECSA-IOL) lineage, collected from 2004 to 2019 and downloaded from the NCBI database (https://www.ncbi.nlm.nih.gov/nucleotide/, accessed on 11 June 2020) according to the following inclusion criteria: (i) being part of the ECSA-Indian Ocean (ECSA-IOL) lineage; (ii) known sampling country; (iii) known sampling year; and (iv) having been already published in peer-reviewed journals. The sequences that did not meet the above reported criteria were excluded.

This dataset was built to study the recent CHIKV epidemics and the distribution of the occurrence of the A226V mutation and other relevant mutations.

The inference of the demographic history and the changes in the effective sample size over time have been also investigated on the CHIKV dataset.

The sampling dates of the dataset ranged from 2004 to 2019. The sampling locations were Sri Lanka (n = 16); Seychelles (n = 1); Mauritius (n = 6); Thailand (n = 26); China (n = 9); India (n = 65); Cambodia (n = 8); Italy (n = 34) and Italy ex-Thailand (n = 2); Singapore (n = 20); Austria ex Thailand (n = 5) and Austria ex-Maldives (n = 1); Israel ex Thailand (n = 1); Slovenia ex Thailand (n = 1); Finland ex Thailand (n = 2); Spain, ex Thailand n = 1; Madagascar (n = 2); Kenya (n = 2); Comoros (n = 3); Reunion (n = 6); Hong Kong (n = 2); Malaysia (n = 11); Bangladesh (n = 11); Pakistan (n = 17); Papua New Guinea (n = 4); and Laos (n = 2).

### 2.2. Phylogenetic Analysis

The sequences of the dataset were aligned using the multiple sequence alignment program MAFFT v.7 [24] under the Galaxy platform [25,26] and manually edited by Bioedit v. 7.0 program [27].

JModeltest [28,29] with the Bayesian Information Criterion (BIC) was used to select the simplest evolutionary model that adequately fitted the sequence data.

The phylogenetic signal in the CHIKV E1 data set of nucleotide sequences was investigated with the likelihood mapping method by analyzing groups of four sequences, randomly chosen, called quartets [30]. For a quartet, just three unrooted tree topologies are possible. The likelihood of each topology is estimated with the maximum likelihood method, and the three likelihoods are reported as a dot in an equilateral triangle (the likelihood map) as already described [30]. If more than 33% of dots falls within the central area indicated, a substantial star-like signal that is a star-like outburst of multiple phylogenetic lineages [30]. Likelihood mapping analyses were performed with the program TREE-PUZZLE [31] for each data set by analyzing 100,000 random quartets.

### 2.3. Evolutionary Rate Estimate, Time-Scaled Phylogeny Reconstruction, and Bayesian Phylogeography

A root-to-tip regression analysis was made using TempEst v.1.5.3 in order to investigate the temporal signal of the dataset [32].

The CHIKV E1 mean evolutionary rate was estimated by using a Bayesian MCMC approach with BEAST v. 1.8.4 [33,34,35] implementing the TN93+I+G model, selected with JModelTest, using both a strict and an uncorrelated log-normal relaxed clock model. As coalescent priors, three parametric demographic models of population growth (constant size, exponential, and expansion growth) and two non-parametric models (the Bayesian skyline plot, BSP, and the Smooth skyride plot Gaussian Markov Random Field—GMRF) were compared. The best fitting models were selected using a Bayes Factor (BF with marginal likelihoods). In accordance with Kass and Raftery [36], the strength of the evidence against H0 was evaluated as follows: 2lnBF < 2, no evidence; 2–6, weak evidence; 6–10, strong evidence; and >10, very strong evidence. A negative value indicates evidence in favor of H0. Only values of >6 were considered significant.

Chains were conducted for at least 500 × 10^6^ generations and sampled every 50,000 steps. The convergence of the MCMC was assessed by calculating the ESS for each parameter. Only parameter estimates with ESSs of >200 were accepted. Maximum clade credibility trees were obtained from the trees posterior distributions with the Tree-Annotator software v 1.8.4, included in the BEAST package [34,35]. Statistical support for specific monophyletic clades was assessed by calculating the posterior probability.

The continuous-time Markov Chain (MCC) process over discrete sampling locations implemented in BEAST [33] was used for the geographical analysis, implementing the Bayesian Stochastic Search Variable Selection (BSSVS) model, which allows the diffusion rates to be zero with a positive prior probability. A comparison of the posterior and prior probabilities of the individual rates being zero provided a formal BF for testing the significance of the linkage between locations. The final trees were manipulated in FigTree v. 1.4 for display. The most probable location of each node was highlighted by labeling the branches with a different color.

### 2.4. CHIKV E1 Mutations

The presence of specific amino acid substitutions, such as A226V, D284E, K211E, K211N, and A98T, was investigated in the CHIKV E1 dataset due to their role as adaptive vector mutations or key signature substitutions [23,37,38]. Based on the phylogeographic tree reconstructed here, we also estimated the time and place of occurrence of the A226V mutation in the CHIKV E1 dataset, reporting the presence of this mutation in the tree Figure.

The identification of the positions of the mutations has been performed with respect to the Reference S27 African prototype (Acc. Number: AF369024).

### 2.5. CHIKV E1 Evolutionary Demographic Reconstruction (Bayesian Skyline Plot)

The population dynamic of the E1 CHIKV dataset was investigated by using the Bayesian Markov Chain Monte Carlo (MCMC) method, implemented in the BEAST software v. 1.8.4 [34,35], with the Bayesian Skyline plot (BSP) demographic model of population growth in order to monitor the changes in the effective number of infections over time.

## 3. Results

### 3.1. Likelihood Mapping

The phylogenetic noise of the data set was investigated by means of likelihood mapping. The tree puzzle likelihood mapping contained 26% unresolved quartets (as shown in Figure S1); as this percentage was not higher than the 33% of dots in the star-like central region, the dataset contained a reliable phylogenetic signal and was suitable for further phylogenetic analysis.

### 3.2. Estimated Rate of CHIKV E1 Gene Evolution, Time-Scaled Phylogeny Reconstruction, and Bayesian Phylogeography

A root-to-tip regression analysis of the temporal signal from the dataset revealed a correlation coefficient of 0.92 and a coefficient of determination [R2] of 0.84, indicating a positive correlation and association between genetic divergence and sampling time, meaning that this dataset appeared to be suitable for phylogenetic molecular clock analysis. The mean evolutionary rate of the CHIKV E1 gene was estimated on the dataset of 258 sequences, as described above. BF analysis showed that the relaxed clock fitted the data significantly better than the strict clock (2lnBF between the strict and relaxed clock was 104.71 in favor of the relaxed clock). Under the relaxed clock, the BF analysis showed that the BSP was better than the other models (2lnBF > 6). The estimated mean value of the CHIKV E1 evolutionary rate for the dataset was 1.313 × 10^−3^ substitution/site/year (95% HPD 8.709 × 10^−4^–1.827 × 10^−3^).

**Figure 1:**
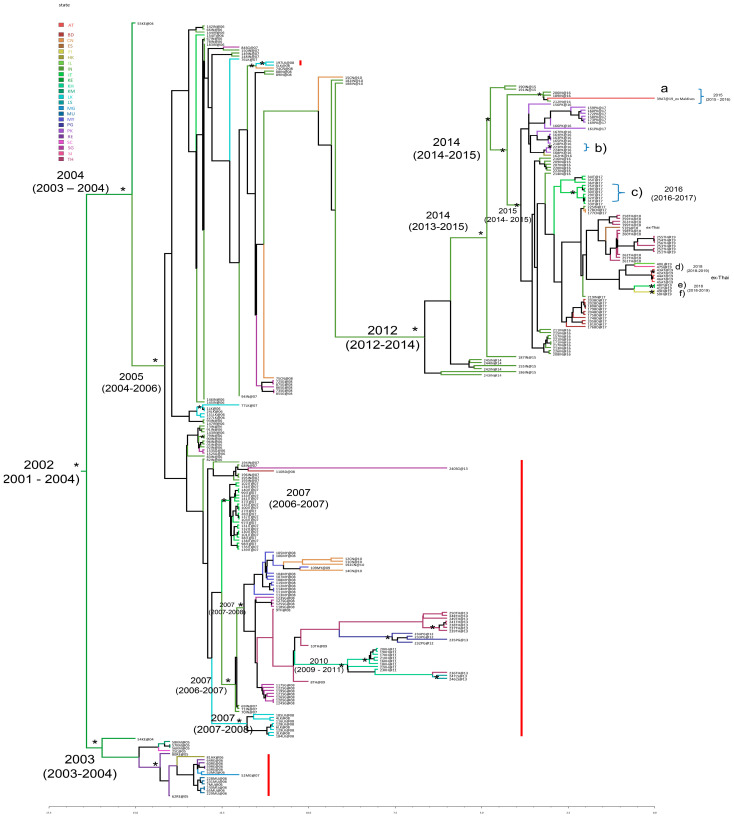
Bayesian phylogeographic tree of CHIKV E1 sequences. The significant statistical support for the clades or clusters subtending the branch is indicated with an asterisk along that branch (posterior probability ≥ 80%). The time of the most recent common ancestor and the credibility interval based on the 95% highest posterior density interval (95% HPD) is reported for the supported nodes. The red bar indicates the presence of the A226V mutation. Geographic locations are shown with different colours in the tree and highlighted in the legend on the left (BD: Bangladesh; CN: China; ES: Spain ex-Thailand; FI: Finland ex-Thailand; HK: Hong Kong; IL: Israel ex-Thailand; IN: India; IT: Italy and Italy ex-Thailand; KE: Kenya; KH: Cambodia; KM: Comoros; LK: Sri Lanka; LS: Laos; MG: Madagascar; MU: Mauritius; MY: Malaysia; PG: Papua New Guinea; PK: Pakistan; RE: Reunion; SC: Seychelles; SG: Singapore; AT: Austria and Austria ex-Thailand; SI: Slovenia ex-Thailand; and TH: Thailand).

Figure 1 showed the Bayesian maximum clade credibility phylogeographic tree on the CHIKV E1 dataset. This analysis showed that the root of the tree most probably originated in 2002 (95% HPD: 2001–2004) in Kenya (state probability, sp = 0.84).

Starting from the tree root, a supported cluster (posterior probability, pp = 0.73) and a significant main clade (pp = 0.82) can be identified. The cluster included mostly isolates from Comoros, Reunion, Mauritius, Madagascar; one strain from Seychelles; and one strain from Hong Kong. The topology of the tree showed a strain isolated in Kenya (state probability, sp = 0.93) as the outgroup for this cluster.

The mean tMRCA estimates for this cluster were 2003 (95% HPD: 2003–2004). The main clade originated in Kenya (sp = 0.96) in 2004 (95% HPD: 2003–2004) and included sequences from India, Singapore, Hong Kong, Sri Lanka, Italy, Bangladesh, China, Pakistan, Malaysia, Thailand (including sequences of cases who acquired the infection in Thailand but were detected in Austria, Israel, Slovenia, Finland, and Italy), Papua New Guinea, Cambodia, and Laos. After the origin in Kenya, the mean tMRCA estimate for the next significant internal node was 2005 (95% HPD: 2004–2006), with the most probable location in India (sp = 0.82). From this node, some supported clusters dating back to 2007 were found (a cluster including Italian strains; a cluster with sequences from Malaysia, China, Singapore, Thailand, Papua New Guinea, Cambodia, and Laos; and a cluster including genomes from Sri Lanka).

The most recent strains (with collection dates from 2014 to 2019) investigated in this study were found located within a supported cluster (pp = 0.88) highlighted in Figure 1. This cluster originated towards mid-2012 (95% HPD: 2012–2014) in India (sp = 0.98) and included sequences from India, Pakistan, Hong Kong, Italy, China, Thailand (including sequences of cases who acquired the infection in Thailand but were detected in Austria, Spain, Israel, Slovenia, Finland, and Italy), and Bangladesh (Figure 1).

The node including the isolates collected from 2016 to 2019 (pp = 0.99) dated back to the end of the year 2014 (95% HPD: 2014–2015) with the most probable location in India (sp = 0.99). The 2017 CHIKV Italian sequences of the outbreak in the Lazio Region, with its secondary outbreak to the South, were located inside a sub-clade of this clade, dating back to 2015 (95% HPD: 2014–2015), originating in India (sp = 0.98) and including strains from different countries (India, China, Thailand, Bangladesh, Pakistan, etc.). Some statistically supported clusters are shown in Figure 1.

Cluster a (pp = 1), including three sequences from India and one from Austria-ex Maldives (39AT@19), dated back to the end of 2015 (95% HPD: 2015–2016), originating in India (sp = 0.99). Cluster b included two isolates from Pakistan dating back to 2016, while cluster c included seven isolates from Italy dating back to the year 2016 (95% HPD: 2016–2017, with the most probable location being Italy sp = 0.99). The other three statistically supported clusters (d, e, and f) can be identified in Figure 1: cluster d (pp = 0.97), including two sequences from Austria ex-Thailand, dated back to the end of the year 2018 (95% HPD: 2018.71–2019); cluster e (pp = 0.89), composed of two sequences from Italy ex Thailand dating back to the end of 2018 (2018.99 with 95% HPD: 2018.53–2019); and the last (pp = 1) including two sequences from Finland ex Thailand also dating back to the end of the year 2018 (95% HPD: 2018–2019). The phylogeographic analysis confirmed the African origin of the CHIKV ECSA genotype, in Kenya. From there, the spread subsequently involved two distinct routes: one throughout the Indian Ocean (Reunion and Comoros) and the other moving towards India; from India, it then scattered in South East Asia, reaching Italy at the end of 2006–beginning 2007 (Figure S2). Then, the circulation continued both from India towards other Asian countries (i.e., Sri Lanka, Singapore, Malaysia, Bangladesh, and China), among Asian countries (i.e., from Malaysia to China, from Singapore to Thailand, from Thailand to Cambodia, from China to India, from Cambodia to Laos, and from Cambodia to Thailand) but also involving other links, i.e., from Thailand to Papua New Guinea (Figure S2).

By analyzing the diffusion flows related to the most recent isolates (the cluster dated back to the end of 2014), the following main links can be highlighted (Figure S2):

- Further expansion in India (years 2014–2015)
- Spread from India to Pakistan (approximately May 2016)
- Spread from Pakistan to Hong Kong (September 2016)
- Spread from India to Thailand (December 2017–September 2019, with multiple cases acquired in Thailand and detected as imported cases in several non-endemic countries, such as Austria, Spain, Italy, Israel, Finland, and Slovenia).

### 3.3. CHIKV E1 Mutations

The presence of specific mutations, such as A226V, D284E, K211E, K211N, and A98T, was investigated in the CHIKV E1 dataset because considered adaptive vector mutations, i.e., those that facilitate transmission by *Ae. albopictus*, or key signature substitutions as described in the Methods section. The most recent CHIKV strains characterized by collection dates ranging from 2014 to 2019, here investigated, did not show the mutation A226V (Figure 1). The presence of A226V in the sequences of the dataset was highlighted in Figure 1 with a red bar and indicated in Table 1. Mutation D284E was present in all strains except one (labeled 51ES@18 with Accession Number: MN080498), showing a gap in that position (Table 1). The amino acid substitution K211E was detected in 37.6% (97/258) of the sequences of our dataset (Table 1). In particular, almost all the recent (collection dates 2014 to 2019) CHIKV strains harbored the mutation K211E, except the sequences labeled with the following codes: 155IN@15 (Accession Number: MF573001.1), 242IN@14 (Accession Number: MN102099.1), 244IN@14 (Accession Number: KX358410.1), and 245IN@14 (Accession Number: KX358408.1). It was present in sequences from different countries (India, Bangladesh, Finland ex Thailand, Italy, Italy ex Thailand, Austria ex Thailand, Singapore, Israel ex Thailand, Thailand, China, Hong Kong, Pakistan, and Spain ex Thailand). Three sequences (1.2%) showed the amino acid substitution K211N (Table 1). Regarding A98T, no sequences showed that mutation; in fact, 219 CHIKV E1 sequences harbored the amino acid alanine (at the a.a. position 98), and 39 sequences showed a gap because the initial portion was missing.

### 3.4. CHIKV E1 Evolutionary Demographic Reconstruction

**Figure 2:**
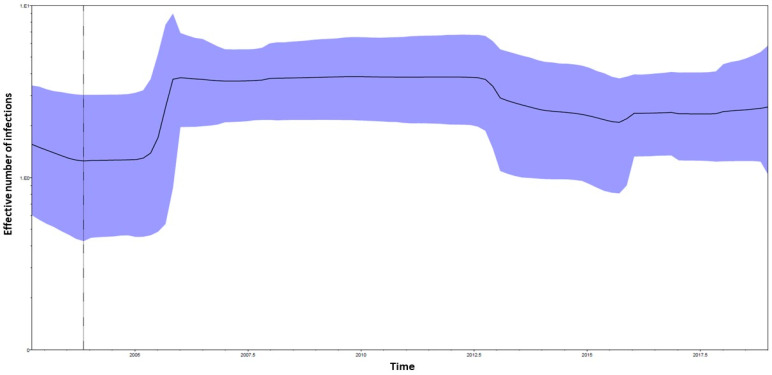
Bayesian skyline plot (BSP) of the CHIKV E1 dataset. The effective number of infections is reported on the *Y*-axis. Time is reported in the *X*-axis. The colored area corresponds to the credibility interval based on 95% highest posterior density interval (HPD). The dotted vertical line is the lower 95% HPD on CHIKV E1 dataset TMRCA.

The Bayesian skyline plot performed on the E1 CHIKV dataset (Figure 2) showed growth in the effective number of the infections between 2005 and until middle 2007. After a constant phase, a mild reduction in the effective number of the infections starting from mid-2012 until 2015 was observed. Starting from 2015, a mild growth of the curve was observed.

## 4. Discussion

CHIKV is considered as a global health threat due to its geographical expansion and adaptation to new vectors [39,40]. Phylogenetic and phylogeographic analysis are essential to track the genetic diversity and circulation of CHIKV. The evidence presented herein offers deepened insight into the phylogenetic relationships and spread of CHIKV, internationally. The CHIKV E1 mean evolutionary rate estimated in this study was 1.313 × 10^−3^ substitution/site/year (95% HPD: 8.709 × 10^−4^–1.827 × 10^−3^), comparable to those reported previously [41,42,43].

The phylogeographic analyses of the CHIKV isolates sampled from different countries permitted the reconstruction of their spread.

In this analysis, the CHIKV strains linked to recent epidemic waves, represented in our dataset and also including the Indian Ocean islands and the Indian subcontinent strains of the years 2004–2007, most likely originated in Kenya in 2002 (95% HPD: 2001–2004), as previously reported [17,43], followed by dissemination to other countries through two distinct routes: one through the Indian Ocean (Reunion, Comoros etc.) and the other through India, and subsequently from India throughout South East Asia, finally reaching Italy in the year 2007 (95% HPD: 2006–2007). The findings estimated through our model are consistent with epidemiological data and with what really happened in that period, i.e., the 2007 Emilia-Romagna (Italy) autochthonous outbreak, when outbreak investigation and surveillance implementation detected more than 200 human cases [2,44,45]. However, the dates of origin here estimated might present some limitations in certain conditions, due to the modeling approach or model parameters that may fail to detect the seasonality of vector-borne infections in temperate climate countries.

The most recent CHIKV strains of our dataset (collected from 2014 to 2019), investigated in this study, were found to be related in a clade originating from India towards mid-2012 (95% HPD: 2012–2014). The 2017 CHIKV Italian sequences of the outbreak in the Lazio Region, with its secondary outbreak in the Calabria Region in southern Italy, were highlighted in a subclade (dating to 2015) of this clade, also derived from India.

The deepening of the phylogenetic relationships, genomic monitoring, and mutation analysis is very important to rapidly detect and characterize CHIKV strains, helping to obtain deeper insights into the evolutionary process and the tracing and management of infections, especially during specific outbreaks, such as that which occurred in Lazio Region (Italy) in 2017, which posed the problem of the risk of re-emergence and endemization in our country, where the competent vector *Ae. albopictus* is largely established [3].

The investigation of the E1 mutations able to increase virus adaptation to *Ae. albopictus* vector is important, since this vector species is rapidly expanding throughout the world [46]. All the recent CHIKV strains here investigated, characterized by collection dates ranging from 2014 to 2019, did not carry the A226V mutation, known to be involved in the increased susceptibility of *Ae. albopictus* for the infection and transmission of CHIKV. Meanwhile, the mutation D284E was observed in all the strains of our dataset except one that was a shorter sequence. Moreover, our data showed that almost all the recent CHIKV strains harbored K211E.

K211E and D284E mutations have been reported previously in Delhi samples [47]. The amino acid position 211 has been suggested to be a site under significant positive selection. The mutation K211E was first described in 2010 in an autochthonous case linked to an imported case from India to southern France, where *Ae. albopictus* is the sole vector [22].

The recent 2017 Italian outbreak strain, vectored by *Ae. albopictus*, not showing the A226V but harboring the K211E mutation, suggests that this mutation can also confer adaptation of the virus to *Ae. albopictus*.

Our study showed that all the sequences of our dataset harbored the amino acid A at position 98, except 39 CHIKV shorter sequences (showing a gap), thus highlighting the absence of the A98T mutation in our dataset. Population dynamic analysis through the Bayesian skyline plot showed a growth trend in the effective number of the infections, compatible with the time periods in which a major number of CHIKV outbreaks and cases occurred, probably also driven by human movements and climate changes.

Further studies are needed to address whether additional mutations can have functional consequences for CHIKV vector/host adaptation, replication, and/or infectivity.

## 5. Conclusions

This study reinforces that continued genomic surveillance, combined with phylogenetic, phylogeographic, and mutation analysis, is needed to support the monitoring of CHIKV evolution and of diversity, and that it could be useful in public health.

Phylogenetic and phylogeographic analysis, even if not directly linked to predictive ability, can be useful for the potential inclusion of the obtained parameters into dedicated species distribution models or global prediction models. In addition, phylodynamic and phylogeographic flows obtained here can represent an archive that can be queried in case of future disease outbreaks.

## Figures and Tables

**Table 1:** Frequency of specific selected mutations (such as A226V, D284E, K211E, K211N, and A98T) considered adaptive vector mutations or key signature substitutions, in the CHIKV E1 dataset (258 sequences).

E1—a.a. Change	n	%
A226V	109	42.2
D284E	257	99.6
K211E	97	37.6
K211N	3	1.2
A98T	/	/

## Data Availability

This study was based on CHIKV sequences available and downloaded from the NCBI database (https://www.ncbi.nlm.nih.gov/nucleotide/).

## Supplementary Materials

The following supporting information can be downloaded at https://www.mdpi.com/article/10.3390/v17030430/s1, Figure S1: Likelihood mapping analysis of CHIKV E1 data set; Figure S2: Phylogeographic reconstruction and dynamics of spatial diffusion of CHIKV E1 sequences.

## Abbreviations

The following abbreviations are used in this manuscript:

CHIKV	Chikungunya virus
ECSA-IOL	Eastern/Central/South African—Indian Ocean Lineage
NSP	Non-structural proteins
MCMC	Markov chain Monte Carlo
BSP	Bayesian skyline plot
GMRF	Gaussian Markov Random Field
BEAST	Bayesian Evolutionary Analysis Sampling Trees
ESS	Effective sample size
BSSVS	Bayesian Stochastic Search Variable Selection
sp	State probability
pp	Posterior probability
HPD	Highest posterior density
