# Supplementary material for: Phylogenetic Analysis of Chikungunya Virus Eastern/Central/South African-Indian Ocean Epidemic Strains, 2004–2019
Source: Viruses. 2025 Mar 18;17(3):430. doi: 10.3390/v17030430 (PMC11945597; doi:10.3390/v17030430)
Supplement: Supplementary file 1 [file viruses-17-00430-s001.zip › viruses-3452251-supplementary.pdf]

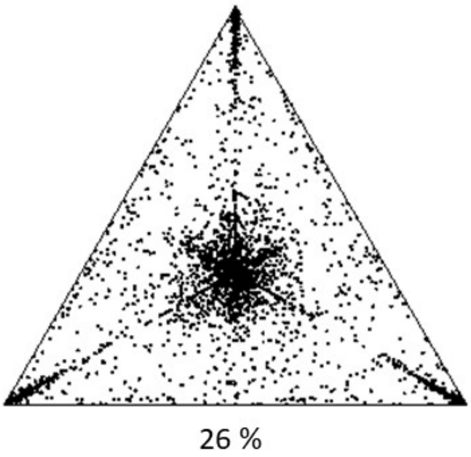

Figure S1. Likelihood mapping analysis of CHIKV E1 data set investigated in this study. The three corners represent fully resolved tree topologies; that is, the presence of tree-like phylogenetic signal in the data. Each dot represents the likelihoods of the three possible unrooted trees for a set of four sequences (quartets) selected randomly from the data set. The central area of the likelihood map represents the star-like signal. At the bottom, the percentage (26%) of unresolved quartets is indicated.

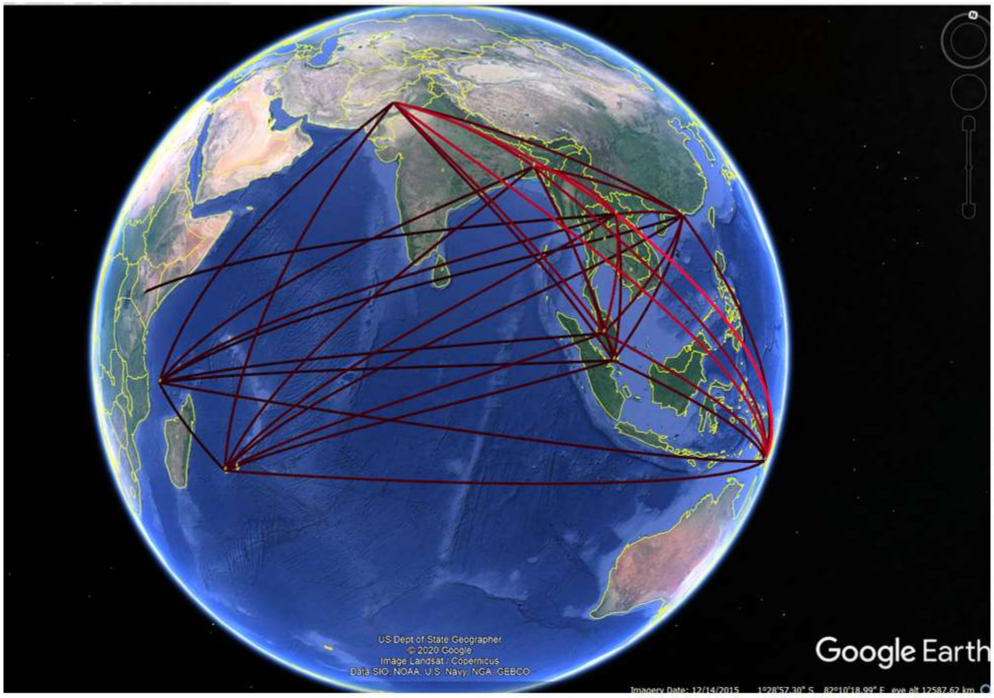

Figure S2. Phylogeographic reconstruction and dynamics of spatial diffusion of CHIKV E1 sequences obtained from the location annotated MCCT. The map was reconstructed with Spread and based on satellite pictures made available in Google Earth (Google Earth Pro V.7.3.2.5491). The main links were reported.
